# A multi-institutional exploration of emergency medicine physicians’ attitudes and behaviours on antibiotic use during the COVID-19 pandemic: a mixed-methods study

**DOI:** 10.1186/s13756-023-01230-2

**Published:** 2023-03-29

**Authors:** Zhilian Huang, Evonne Tay, Win Sen Kuan, Ling Tiah, Yanyi Weng, Hann Yee Tan, Eillyne Seow, Li Lee Peng, Angela Chow

**Affiliations:** 1 Department of Preventive and Population Medicine, Office of Clinical Epidemiology, Analytics, and Knowledge [OCEAN], Tan Tock Seng Hospital, 11 Jalan Tan Tock Seng, Singapore, 308433 Singapore; 2 Department Emergency Medicine, National University Hospital, Singapore, Singapore; 3 Department of Surgery, Yong Loo Lin School of Medicine, National University of Singapore, Singapore, Singapore; 4 Accident & Emergency Department, Changi General Hospital, Singapore, Singapore; 5 Department Emergency Medicine, Tan Tock Seng Hospital, Singapore, Singapore; 6 Acute and Emergency Care Department, Khoo Teck Puat Hospital, Singapore, Singapore; 7 Lee Kong Chian School of Medicine, Nanyang Technological University, Singapore, Singapore; 8 Infectious Diseases Research and Training Office, National Centre for Infectious Diseases, Singapore, Singapore; 9 Saw Swee Hock School of Public Health, National University Singapore, Singapore, Singapore

**Keywords:** Antimicrobial resistance, Antibiotics, Emergency department, COVID-19

## Abstract

**Background:**

The COVID-19 pandemic has changed the epidemiology of upper respiratory tract infections (URTI) and the disease profile of patients attending the emergency department (ED). Hence, we sought to explore the changes in ED physicians’ attitudes and behaviours in four EDs in Singapore.

**Methods:**

We employed a sequential mixed-methods approach (quantitative survey followed by in-depth interviews). Principal component analysis was performed to derive latent factors, followed by multivariable logistic regression to explore the independent factors associated with high antibiotic prescribing. Interviews were analysed using the deductive-inductive-deductive framework. We derive five meta-inferences by integrating the quantitative and qualitative findings with an explanatory bidirectional framework.

**Results:**

We obtained 560 (65.9%) valid responses from the survey and interviewed 50 physicians from various work experiences. ED physicians were twice as likely to report high antibiotic prescribing rates pre-COVID-19 pandemic than during the pandemic (AOR = 2.12, 95% CI 1.32 to 3.41, *p* = 0.002). Five meta-inferences were made by integrating the data: (1) Less pressure to prescribe antibiotics due to reduced patient demand and more patient education opportunities; (2) A higher proportion of ED physicians self-reported lower antibiotic prescribing rates during the COVID-19 pandemic but their perception of the overall outlook on antibiotic prescribing rates varied; (3) Physicians who were high antibiotic prescribers during the COVID-19 pandemic made less effort for prudent antibiotic prescribing as they were less concerned about antimicrobial resistance; (4) the COVID-19 pandemic did not change the factors that lowered the threshold for antibiotic prescribing; (5) the COVID-19 pandemic did not change the perception that the public's knowledge of antibiotics is poor.

**Conclusions:**

Self-reported antibiotic prescribing rates decreased in the ED during the COVID-19 pandemic due to less pressure to prescribe antibiotics. The lessons and experiences learnt from the COVID-19 pandemic can be incorporated into public and medical education in the war against antimicrobial resistance going forward. Antibiotic use should also be monitored post-pandemic to assess if the changes are sustained.

**Supplementary Information:**

The online version contains supplementary material available at 10.1186/s13756-023-01230-2.

## Introduction

Antimicrobial resistance (AMR) occurs when changes in microorganisms render the drugs used to treat infections ineffective. The World Health Organization projected that AMR infections will cause 10 million deaths annually by 2050 [1]. These infections also result in more outpatient visits, excess length of stay in hospitals, and increased utilization of intensive care and isolation units [2].

Antimicrobial stewardship, a coordinated program that promotes the appropriate use of antimicrobials, has reduced unnecessary antibiotic use in inpatient settings [3, 4]. Despite the widespread implementation of antimicrobial stewardship programs by hospitals, the prevalence of multi-drug resistant organisms has increased in recent years [5, 6], often due to the liberal use of antibiotics in ambulatory settings [7]. Antimicrobial stewardship efforts have often been neglected in the emergency department (ED) [8]. Prior to the coronavirus disease 2019 (COVID-19) pandemic, upper respiratory tract infections (URTI) accounted for 20–25% of ED visits [9, 10]. These ED visits are often unnecessary as they can be easily managed in the primary care setting. Furthermore, up to a third of uncomplicated URTI visits to the ED results in an antibiotic prescription, which is often unwarranted [11, 12]. Uncomplicated URTI visits to the EDs are often unnecessary as they can be easily managed in the primary care setting. Factors influencing antibiotic prescribing in the ED include perceived patient expectations for antibiotics, diagnostic uncertainties, socio-demographic factors of the patient, time pressure faced in the ED, influence from colleagues, and personal experience in practicing medicine [13–15].

There was a slight increase in antibiotic use and the use of broad-spectrum antibiotics during the early days of the COVID-19 pandemic [16]. The initial uncertainties of the disease and a surge in COVID-19 related caseloads prompted physicians to prescribe more (and broad-spectrum) antibiotics to prevent secondary bacterial infections [16–18]. Antibiotic prescribing behaviours were subsequently normalised with a better understanding of COVID-19. Furthermore, infection prevention and control measures in response to the COVID-19 pandemic (i.e., mask-wearing, social distancing) have inadvertently altered the epidemiology of URTIS [19] and could have recalibrated a ‘new normal’ for antibiotic use in the ED.

The attitudes and behaviours of antibiotic use among ED physicians would have changed during the COVID-19 pandemic due to changes in the profile of ARI patients attending the EDs. These changes present a golden opportunity to enhance antimicrobial stewardship efforts in the ED, which is often less well prioritised due to time constraints and workloads. We employed a novel mixed-methods approach to explore ED physicians’ attitudes and behaviours towards antibiotic prescribing in four EDs in Singapore, prior to and during the COVID-19 pandemic, and made meta-inferences to guide future efforts to address AMR.

## Methods

### Overview

We took a pragmatic stance and employed an explanatory sequential mixed-methods approach to assess the changes in attitudes and behaviours of ED physicians towards antibiotic use for uncomplicated upper respiratory tract infection (URTI) during the COVID-19 pandemic. The data collection for the quantitative phase (a cross-sectional survey) commenced prior to the COVID-19 pandemic to assess the attitudes and behaviours of ED physicians towards antibiotic prescribing for URTI to guide the design of targeted antimicrobial stewardship interventions in the ED. However, the protracted COVID-19 pandemic shifted the local epidemiology of ARIs, altered patients’ health-seeking behaviours, and changed the practice norms of physicians in the ED. These changes provided us with an opportunity to compare the attitudes and behaviours of ED physicians regarding antibiotic prescribing prior to and during the COVID-19 pandemic. We first conducted a quantitative survey and used its preliminary findings to design the interview guide for the qualitative study. The quantitative data collection occurred from November 2019 to December 2021, while the qualitative data collection occurred from May 2021 to September 2021.

### Participants

Participants comprise physicians who were working at one of the four adult EDs in four acute care hospitals (Changi General Hospital, Khoo Teck Puat Hospital, National University Hospital and Tan Tock Seng Hospital) in Singapore at the point of the survey or in-depth interview (IDI). All physicians were invited to participate in the questionnaire survey, whilst junior and senior physicians with a wide range of working experience in the EDs were purposively sampled for the IDIs to ensure the maximum variation of views.

### Survey questionnaire

The survey questionnaires comprise 23 and 29 five-point Likert-scale items measuring physicians’ attitudes and behaviours, respectively towards antibiotic prescribing for URTI (Additional file 1: Tables S1a and S1b). The attitude-related items were scored on a five-point Likert agreement scale (i.e., Strongly agree, Agree, Neutral, Agree, Strongly Disagree) while the behaviour-related items were scored on a five-point Likert frequency scale (i.e., Always, Most of the time, Sometimes, A few times, Never). The questions were revised from a previous survey [20] and constructed with reference to the literature on the factors influencing physicians’ antibiotic prescribing for URTI [14, 15, 21]. In our case, URTI refers to patients having symptoms of acute nasopharyngitis, sinusitis, pharyngitis, tonsillitis, laryngitis, tracheitis, and epiglottitis. We also collected information on the physicians’ occupational background (i.e., designation, years of experience, place of medical education), the self-reported proportion of URTI patients they had medically attended to, and the proportion of patients for whom they prescribed antibiotics.

### Quantitative analysis

The primary outcome of interest was the respondents’ self-reported proportion of URTI patients for whom antibiotics were prescribed. A high antibiotic prescribing rate was defined as having a reported antibiotic prescribing rate of > 10% of the URTI patients [22].

Principal component analysis (PCA) was performed to derive latent factors from the attitude- and behavioural-related questions. We used Horn’s parallel analysis to determine the optimal number of factors for dimension reduction [23]. Likert items were removed stepwise by optimizing the overall variance explained (desirable to be higher) and internal consistency between the factors. Internal consistency was measured by Cronbach’s alpha of each factor (> 0.7 is considered good). We conducted non-parametric tests (Chi-square for categorical variables and Mann–Whitney U for continuous variables) to assess for statistical differences between high and moderate prescribers at baseline (Table 1).

**Table 1:** Baseline characteristics of quantitative survey participants

Baseline characteristics of respondents, *n* (%)	Moderate antibiotic prescriber (N = 380)	High antibiotic prescriber (N = 177)	*P*-value
*Institution*			
Institution 1	67 (17.6%)	14 (7.9%)	0.001*
Institution 2	74 (19.5%)	56 (31.6%)	
Institution 3	99 (26.1%)	42 (23.7%)	
Institution 4	140 (36.8%)	65 (36.7%)	
*Years of experience*	(N = 377)	(N = 177)	
≤ 5 Years	56 (14.9%)	45 (25.4%)	0.003*
*Basic medical education*	(n = 378)	(N = 177)	
Singapore	228 (60.3%)	100 (56.5%)	0.394
*COVID-19 status*	(n = 380)	(n = 177)	
Pre-DORSCON Orange	126 (33.2%)	129 (73.3%)	0.001*
*Physician Designation*	(n = 376)	(n = 176)	
Junior physicians^#^	302 (80.3%)	129 (73.3%)	0.063
*Attitude factor scores, Mean (SD)*	(n = 377)	(n = 177)	
Factor 1: Perception of antibiotic over-prescribing in the ED	− 0.160 (0.98)	0.351 (0.95)	< 0.001**
Factor 2: Perception of organization safety culture in the ED	0.054 (0.99)	− 0.129 (1.00)	0.114
Factor 3: Perception of the utility of clinical decision support tools for antibiotic prescribing	− 0.022 (1.04)	0.050 (0.92)	0.888
Factor 4: Perception of the utility of patient education on antibiotics use and antibiotics resistance	− 0.021 (1.00)	0.039 (1.01)	0.449
Factor 5: Insufficient patient education	− 0.004 (1.03)	0.018 (0.94)	0.776
*Behaviour factor scores, Mean (SD)*	(n = 370)	(n = 175)	
Factor 1: Pressure to prescribe antibiotics (Patient attributed)	− 0.213 (0.81)	0.456 (1.20)	< 0.001**
Factor 2: Effort to prescribe antibiotics prudently due to concerns about antibiotic resistance	0.179 (0.96)	− 0.379 (0.98)	< 0.001**
Factor 3: Lowered threshold for antibiotics prescribing	− 0.241 (0.98)	0.524 (0.83)	< 0.001**
Factor 4: Peer influence on antibiotic prescribing	0.055 (0.99)	− 0.138 (1.00)	0.002*

DORSCON, Disease Outbreak Response System Condition; ED, emergency department; SD, standard deviation

Chi-Square and Mann–Whitney U tests were conducted for categorical and continuous variables respectively

^#^Junior physicians refer to Medical Officers, Residents, Resident Physicians, Senior Resident Physicians. All other ranks were classified as senior physicians

^*^p < 0.05

^**^p < 0.001

Finally, we explored the independent factors associated with high antibiotic prescribing using multivariable logistic regression. The five attitude-related factors, four behaviour-related factors, institution, physician seniority by designation (binary), place of medical school (binary), years of medical practice (binary—more or less than five years), and data collection period (binary—pre or post COVID-19), were included in the initial logistic regression model. We defined the cutoff of pre and post COVID-19 as 7th February 2020 when Singapore raised its risk alert level to Disease Outbreak Response System Condition (DORSCON) Orange (moderate disruptions to daily life with the implementation of quarantine, temperature screening, and visitor restrictions at hospitals) [24]. We further included interaction terms between pre-/post- COVID-19 pandemic and all the nine factors derived from the PCA in the initial model to explore the modifying effect of the COVID-19 pandemic on antibiotic prescribing for URTI.

Variables in the initial model were first removed in a stepwise manner based on the likelihood ratio tests and Akaike’s information criterion (AIC). The items that were dropped from the PCA were then added as variables to the model if adding the item reduces the AIC. The model with the lowest AIC was selected as the final model (Additional file 1: Table S2). Statistical significance was set at *P* < 0.05, and all statistical assumptions were checked to ensure the accuracy of the analyses. IBM SPSS Statistics for Windows, version 26 (BM Corp., Armonk, NY, USA) [25] was used for the statistical analyses.

### Qualitative interview procedure

We piloted the interview guide with three ED physicians to refine the interview process. All IDIs were conducted over zoom, audio recorded, and transcribed verbatim. Up to 15 respondents, ranging from medical officers to senior consultants, were purposively sampled within each institution to ensure maximum variation. Each interview lasted for approximately 40 min and involved at least one notetaker. The interviewers (ET and KN) took turns to conduct the one-on-one in-depth interviews and take notes while calibrating the interviews. After the interviews, the notetaker(s) would progressively fill up a broad framework matrix based on the socio-ecological model (SEM) to assess for data saturation.

The interviews were semi-structured, where participants were asked open-ended questions from a guide, followed by probes to explore and deepen the understanding of a discussion topic (Additional file 1: Table S3). We first asked participants about their definitions of URTI and what they thought was the usual practice for treating URTI in the ED prior to the COVID-pandemic. Next, we asked them about their experiences in URTI management and the use of antibiotics for URTI pre and during the COVID-19 pandemic. The discussion on experiences focused on the factors that influence physicians’ antibiotic prescribing practices, such as patient-provider relationship, peer influence, departmental practices, diagnostics, and patient factors. Finally, we assessed participants’ attitudes toward AMR and asked them for suggestions to improve antimicrobial stewardship efforts in the ED.

### Qualitative analysis

Three study team members (ET, KN, ZH), who were interviewers and notetakers of the IDIs, coded the qualitative data using the deductive-inductive-deductive Framework method [26]. Data familiarization was achieved through notetaking during the interview and review of the outsourced transcripts. After data familiarization, all team members independently conducted open coding on one transcript using a broad deductive approach. Important categories were initially included, and coders could add or collapse categories during the open coding process. Team meetings were convened to calibrate new broad categories for the next calibration exercise. Cohen’s Kappa was used to calculate the coding agreement between two coders. The coding calibration exercise covered transcripts from various physician designations and institutions and was repeated until we achieved an average Kappa score of > 0.7. The remaining transcripts were then distributed to study team members to code independently. The team further discussed new categories that arose at the end of the coding exercise (i.e., after coding all the transcripts).

After finalizing the coding categories, ET and ZH reviewed all the data in the categories, collapsed and renamed some categories, and recoded some of the codes. The coded data were then charted into a framework matrix (by institution and physician seniority) for analysis and interpretation. The final analysis was not anchored on the SEM framework, although the broad deductive approach was guided by it initially. NVivo 12 Pro was used to analyse the qualitative data [27]

### Integrating qualitative and quantitative results

We employed the explanatory bidirectional framework by Moseholm et al. to merge the qualitative and quantitative results [28]. This approach initially uses the quantitative domain to search the qualitative data and subsequently uses the themes emerged from the qualitative data to corroborate with findings from the quantitative data.

We first considered the factors included in the final logistic regression model as initial codes to search for similar themes emerging from the qualitative analysis. These analyses were juxtaposed to form preliminary meta-inferences. Next, we looked for possible logical links between the attitude-related Likert-scale items with the behaviour-related factors in the final logistic regression model to substantiate the meta-inferences. Although the directional links between attitudes and behaviours have been established, studies have demonstrated the inter-dependency between the two attributes [29]. We also considered the quantitative model building process by removing the factor interaction terms as a factor remaining status quo pre- and during-COVID-19 pandemic. We then delved into the descriptive analyses of individual Likert-scale items to substantiate themes that emerged later from the qualitative analyses. Finally, the preliminary meta-analyses were refined through various data iterations to derive the most logical meta-inference.

## Results

### Quantitative phase

We invited 850 physicians to participate in the study and collected 560 (65.9%) valid survey responses (Institution 1: 83/181 (45.9%), Institution 2: 130/167 (77.8%), Institution 3:142/234 (60.7%), Institution 4: 205/268 (76.5%)). Of these responses, three had the outcome (i.e., the percentage of antibiotics prescribed for URTI) and 23 had other variables missing. Cases with missing variables were dropped from the final model (4.6% (26/560)). We obtained five latent factors related to the antibiotic prescribing attitudes of the physicians and four latent factors related to the antibiotic prescribing behaviour of the physicians from the PCA (Additional file 1: Tables S1a and S1b, Table 1).

#### Baseline characteristics of participants

Overall, 31.8% (177/557) of respondents reported high antibiotic prescribing rates (prescribing in > 10% of URTI patients). Institution 1 had a lower proportion of high antibiotic prescribers (7.9% vs. 17.6%) while institution 2 had a higher proportion of antibiotic prescribers (31.6% vs. 19.5%) compared with moderate prescribers in the same institution (p = 0.001). A higher proportion of high antibiotic prescribers had five or less years of work experience (25.4% vs. 14.9%, *p* = 0.003), and answered the survey pre-COVID-19 pandemic (73.3% vs. 33.2%, *p* < 0.001). We also observed statistically significant differences in some of the attitudinal and behavioural factors between moderate and high antibiotic prescribers. Higher mean scores indicate higher perception of an attitude or a high tendency to exhibit a behaviour. High prescribers had a higher perception that antibiotics were over-prescribed in the ED (*p* < 0.001), had a higher tendency to succumb to patient attributed pressure to prescribe antibiotics (*p* < 0.001) and had a lower tendency to prescribe antibiotics prudently due a lack of concern about antibiotic resistance (*p* < 0.001). High prescribers also had a higher tendency to lower their threshold for antibiotic prescribing (*p* < 0.001) and a lower tendency to be influenced by their peers (*p* = 0.002). We did not observe any statistically significant differences in the country of medical education and physician designation between moderate and high prescribers (Table 1).

#### Characteristics of antibiotic prescribing pre and post COVID-19

The final multivariable logistic regression model included 12 variables and two interaction terms (Table 2). Respondents in the pre-COVID-19 pandemic period were twice as likely to report high antibiotic prescribing rates than respondents during the pandemic [adjusted odds ratio (AOR) = 2.12, 95% confidence interval (CI) 1.32 to 3.41, *p* = 0.002].

**Table 2:** Multivariable analysis of factors associated with high antibiotic prescribing rate for upper respiratory tract infection patients

Model variables	Adjusted model		
	Adjusted OR (95% CI)	*P*-value	VIF
COVID-19 status (pre-DORSCON Orange)	2.12 (1.32, 3.41)	0.002*	1.08
*Attitude-related factors and Likert items*			
Factor 1: Perception of antibiotic over-prescribing in the ED	1.19 (0.92, 1.54)	0.184	1.297
Likert item C1: I am confident of my antibiotic prescribing decisions for patients with URTI	0.70 (0.47, 1.03)	0.067	1.216
Likert item C2: Patients should take antibiotics for URTI because the risks (e.g., drug allergy, side effects etc.) are low	**2.08 (1.39, 3.11)**	< 0.001**	1.283
Likert item C3: Patients attending this ED are demanding about their treatment (including requesting for antibiotics for URTI)	**1.44 (1.09, 1.89)**	0.01*	1.246
Likert item C13: Educating the public on appropriate antibiotic use will reduce my work pressure in handling URTI patients	**1.46 (1.05, 2.02)**	0.025*	1.117
*Behaviour-related factors and Likert items*			
Factor 1: Pressure to prescribe antibiotics (pre-COVID-19)	**1.74 (1.12, 2.71)**	0.014*	4.207
Factor 1: Pressure to prescribe antibiotics (post-COVID-19)	0.99 (0.70, 1.41)	0.973	
Factor 1 interaction (Factor 1 * COVID-19)	**0.57 (0.34, 0.95)**	0.030*	3.217
Factor 2: Effort to prescribe antibiotics prudently due to concerns about antibiotic resistance (pre-COVID-19)	1.10 (0.73, 1.66)	0.645	3.421
Factor 2: Effort to prescribe antibiotics prudently due to concerns about antibiotic resistance (post-COVID-19)	**0.65 (0.46, 0.92)**	0.015*	
Factor 2 interaction (Factor 2 * COVID-19)	**0.59 (0.35, 0.99)**	0.046*	3.121
Factor 3: Lowered threshold for antibiotics prescribing	**1.83 (1.37, 2.42)**	< 0.001**	1.35
Likert item D16: I will prescribe antibiotics for URTI patients to keep on standby, in case they need them later (e.g., travelling to another country, condition deteriorates)	1.25 (0.95, 1.64)	0.109	1.329
Likert item D22: I will prescribe antibiotics for URTI patients, if they have waited a long time for medical consultation	0.90 (0.50, 1.60)	0.708	1.699
Likert item D27: I will discuss with the senior doctors in the department, if I am uncertain about the management of an URTI patient	0.97 (0.79, 1.19)	0.743	1.087

Bold means that the p-value is statistically significant at *p* < 0.05

DORSCON, Disease Outbreak Response System Condition; ED, emergency department; URTI, upper respiratory tract infection; VIF, variance inflation factor

^*^p < 0.05

^**^p < 0.001

#### Physicians' attitude toward antibiotic prescribing

After model adjustments, the perception that antibiotics were over-prescribed in the ED was no longer significantly different between moderate and high antibiotic prescribers. Although the confidence in antibiotic prescribing decisions for patients with URTI was not significantly different between high and moderate antibiotic prescribers, high prescribers were 1.4 times less confident of their prescribing decisions than moderate antibiotic prescribers (AOR = 1.44, 95% CI 0.60 to 1.37, *p* = 0.067). High prescribers were twice as likely to perceive that the risks of antibiotic usage are low (AOR = 2.08, 95% CI 1.39 to 3.11, *p* < 0.001). They were also more likely to perceive patients attending the ED to be demanding (AOR = 1.44, 95% CI 1.10 to 1.89, *p* = 0.01 and that educating patients about antibiotic use will reduce their work pressure in handling patients with URTI (AOR = 1.46, 95% CI 1.05 to 2.02, *p* = 0.025).

#### Physicians’ behaviour toward antibiotic prescribing

Two behaviour-related factors (“Pressure to prescribe” and “Effort to prescribe prudently”) had significant interactions with the (pre/during) COVID-19 pandemic variable. High antibiotic prescribers were more likely to succumb to patient attributed pressure to prescribe antibiotics prior to the COVID-19 pandemic (AOR = 1.74, 95% CI 1.12 to 2.71, *p* = 0.014), but not so during the pandemic (AOR = 0.99, 95% CI 0.698 to 1.41, *p* = 0.973).There was no significant difference in the effort to prescribe antibiotics prudently between high and moderate antibiotic prescribers pre-COVID-19 pandemic (AOR = 1.10, 95% CI 0.73 to 1.66, *p* = 0.6345). However, high prescribers were less likely to make an effort to prescribe antibiotics prudently during the COVID-19 pandemic (AOR = 1.54, 95% CI 1.09 to 2.18, *p* = 0.015).

High antibiotic prescribers were 1.8 times more likely to lower their antibiotic prescribing thresholds for immunocompromised patients, the elderly, patients with a borderline bacterial infection, and patients who had reattended the ED, regardless of the COVID-19 pandemic. The remaining three behaviour-related Likert items (i.e., prescribing antibiotics for standby, prescribing antibiotics for patients with a long waiting time, and discussing with senior doctors uncertain URTI cases) included in the model were not significantly different between moderate and high antibiotic prescribers.

### Qualitative phase

#### Participant demographics

We interviewed 50 physicians with a variable distribution of age, working experience, and designation across the four institutions (Table 3). Participants included medical officers, residents, resident physicians, registrars, and consultants. Hence, we were able to get a wide range of views from physicians with varying experiences in managing URTI patients.

**Table 3:** Baseline characteristics of participants in the qualitative phase

Demographic characteristics	No. of participants (N = 50)
*Age*	
Median (Min, Max)	32.5 (25, 56)
*Years of practice as a physician, n (%)*	
2–5	11 (22)
5–9	21 (42)
≥ 10	18 (36)
*Years of practice in the current emergency department, n (%)*	
< 2	11 (23)
2–5	12 (9)
5–10	15 (7)
≥ 10	12 (11)
*Sex, n (%)*	
Male	28 (56)
Female	22 (44)
*Basic medical education, n (%)*	
Singapore	25 (50)
Overseas	25 (50)
*Postgraduate Education, n(%)*	
Yes	30 (60)
*Professional designation, n(%)*	
Medical Officers/ Residents	16 (32)
Senior Residents/Registrars	8 (16)
Resident Physicians/ Senior Resident Physicians/ Principal Resident Physicians	10 (20)
Associate Consultants	5 (10)
Consultants/ Senior Consultants	11 (22)
*Institutions, n (%)*	
Institution 1	15 (30)
Institution 2	9 (18)
Institution 3	14 (28)
Institution 4	12 (24)

#### Changes in antibiotic prescribing practices prior to and during the COVID-19 pandemic

Since the perception of antibiotic prescribing during the COVID-19 pandemic varied, we grouped the qualitative analyses into four broad themes, (1) Increase in antibiotic prescribing, (2) Acceleration of the development of antibiotic resistance, (3) Decrease in antibiotic prescribing, and (4) No change in antibiotic prescribing. Each of the four themes were broken down into sub themes to explore the changes in physicians’ perception on antibiotic prescribing prior to and during the COVID-19 pandemic. Table 4 shows the summary of the broad themes, sub themes, and representative quotes corresponding to each of the sub themes.

**Table 4:** Broad and subthemes derived from the qualitative analyses of changes in the attitudes and behaviour of antibiotic prescribing for URTI during COVID-19

Broad themes	Sub themes	Examples
Increase in antibiotic prescribing	More X-rays will pick up more specifics	*“When they [the patients] do come in, we are over ordering X-rays, and sometimes the X-ray will describe this equivocal finding, and we will just give antibiotics.” (Institution 2, Medical Officer)* *“Maybe twice the number of people [are] getting chest X-rays than they used to, you pick up a lot more of these non-specific findings, when actually they didn’t need the chest X-ray in the first place. And then when people [physicians] see this, half the time, even if you [patient] don’t have a cough or runny nose, some people [physicians] will just give antibiotics and then say, “oh okay, maybe cover for pneumonia” for someone who just came in with giddiness and lethargy, no fever, no cough, no runny nose. Just empirical cover.” (Institution 3, Senior Resident)*
	Diminished prescribing threshold for prolonged COVID-19 symptoms	*“Before COVID[-19], people do not pay much attention about the respiratory tract infection- respiratory symptoms. So, they just stay at home and take some antipyretics and some symptomatic medication by themselves. After COVID[-19], because they are very afraid of getting infected by COVID[-19] and they are aware of all these symptoms, most of the patients come to the ED after [prolonged] condition. I think most of the patients come to the ED need antibiotics because they are very afraid of these symptoms.” (Institution 1, Resident Physician)* *“Especially the outpatient setting, when we have COVID[-19] positive patients and patients with a poor premorbid that are immunocompromised. We can imagine that in an outpatient setting, the doctors will be worried about possible bacterial infections manifesting themselves in the first few days of symptoms. Therefore, prescribing antibiotics.” (Institution 1, Medical Officer)*
	Less time to explain to patients during a busy shift	*If we end up X-raying more people and there are more equivocal reports coming out, if the condition arises and if it’s a busy shift, we hardly have any time to make a shared decision making with the patient, then I may just succumb to giving[antibiotics].” (Institution 4, Associate Consultant)*
Acceleration of the development of antibiotic resistance	Protocol—To treat COVID-19 pneumonia with antibiotics	*“I think unfortunately because any pneumonia from ED is admitted. When they are admitted, we would end up having to treat—or at least our protocol is that we will end up treating for possible underlying bacterial pneumonia rather than a COVID[-19] related pneumonia, so we do start antibiotics. Given how long this pandemic is stretching out, I’m sure eventually some sort of antibiotic resistance will happen.” (Institution 4, Resident)* *"Okay maybe COVID[-19] cause more URTI, and people- there is a chance of misusing antibiotics. Maybe higher because the increase in the proportion of URTI patient[s]. So, I think that is probably one of the reason why antibiotic resistance lies from there." (Institution 2, Resident Physician)*
	Less priority on the development of new antibiotics	*“All the pharmaceutical companies are trying to ramp up COVID[-19] production, and COVID[-19] vaccine production […], and the research and development into new antibiotics to tackle the problems of antibiotic resistance maybe pushed further down the line.” (Institution 4, Senior Consultant)*
Decrease in antibiotic prescribing	Less consults for non-COVID-19 related URTI complaints	*“I would say probably- probably lesser antibiotic prescription now, based on the experience for the last year. Since we are concentrating more on COVID[-19]. I would say like antibiotics are less prescribed for URTI.” (Institution 1, Staff Registrar)* *“I think the goal of them coming to ED also have shifted, more on they wanted to get a swab test done. So, they don’t really ask for antibiotics.” (Institution 4, Senior Resident Physician)*
	An opportunity to educate patients that antibiotics are not necessary for a viral illness	*“The truth is during this pandemic, we have even more reason to tell them that antibiotic[s] are unnecessary. If you come pre-pandemic, we are not going to do an X-ray for you. We are not going to do a blood test for you. We are not going to do any kind of swab for you. It’s just based on physical exam, and we send them on their way. So now when you come during the pandemic, we have a much lower threshold for doing X-ray and bloods and of course we have to do the COVID[-19] swab. And so, I find it easier to tell patients “You know what, we have done some test[s]. There is really nothing serious and there is really no need for antibiotics”” (Institution 1, Medical Officer)* *I think COVID has taught a lot of people [physicians] that you know it is a viral illness and you don’t go around recommending antibiotics. Starting that piece of knowledge has actually gone out to a lot of common folks, common people in the street. When previously this is a piece of knowledge that they don’t know, [have] never bothered to know.” (Institution 4, Senior Consultant)*
No change in antibiotic prescribing	COVID-19 does not change the standard management of URTI	*I think in general, there’s no shift in the management for uncomplicated cases. Firstly, even for COVID[-19], there’s no [need] for antibiotics. So, we still give the patient symptomatic medications. (Institution 3, Resident)* *Regardless of the COVID[-19] situation, regarding antibiotic practices, I don’t think there’s been much of a change. I think most people still- won’t prescribe antibiotics if they don’t think so.” (Institution 3, Senior Resident)*
	Baseline antibiotic prescribing was low prior to COVID-19	*[In] the ED setting, I don't think it makes much of difference, because COVID[-19] or not COVID[-19], if you should think it's a URTI, you wouldn't be giving antibiotics anyway. (Institution 4, Resident)* *I think it (prescribing practices) remains the same. Even more so now because covid is a viral thing right? So, I feel that my colleagues and I feel the same way. A lot of ARI, [are] predominantly viral in nature [and] we really do not have the habit to prescribe antibiotics for such cases. (Institution 3, Senior Consultant)* *“I mean COVID[-19] is a viral illness. I don't think antibiotics has anything to, as in I mean antibiotics don't treat COVID[-19]. So, it wouldn't change.” (Institution 2, Resident)*

### Increase in antibiotic prescribing

Three subthemes, (1) More X-rays will pick up more specifics, (2) Lowered prescribing threshold for prolonged COVID-19 symptoms, (3) Less time to explain to patients during a busy shift, emerged from the perception of increased antibiotic prescribing in the ED during the COVID-19 pandemic.

#### Subtheme (1): more X-rays will pick up more specifics

During the COVID-19 pandemic, the Singapore Ministry of Health’s guidelines required all ED attendances for ARIs (including URTI) to be supplemented with chest X-rays to rule out suspects of COVID-19. Hence, physicians across various designations and institutions synonymously felt that the additional increase in radiologic investigations during the pandemic could have picked up equivocal findings that would warrant antibiotics.

#### Subtheme (2): lowered prescribing threshold for prolonged COVID-19 symptoms

Some physicians felt that if the COVID-19 infection was severe, physicians would be more inclined to prescribe antibiotics as a precaution against secondary bacterial infection. Physicians would also be more likely to lower their threshold of antibiotic prescribing for patients with prolonged URTI symptoms or COVID-19 patients who are immunocompromised if the condition warranted an ED visit.

#### Subtheme (3): less time to explain to patients during a busy shift

The EDs in Singapore experienced bouts of attendance surges during the COVID-19 pandemic. Hence, one physician mentioned that there could be less time for shared decision making if the X-ray reports picked up equivocal findings.

“If we end up X-raying more people and there are more equivocal reports coming out, if the condition arises and if it’s a busy shift, we hardly have any time to make a shared decision making with the patient, then I may just succumb to giving[antibiotics].” (Institution 4, Associate Consultant).

### Acceleration of the development of antibiotic resistance

Two subthemes, (1) Protocol—To treat COVID-19 pneumonia with antibiotics, (2) Less priority on the development of new antibiotics, emerged from the perception that the COVID-19 pandemic would accelerate the development of antibiotic resistance.

#### Subtheme (1): protocol to treat COVID-19 pneumonia with antibiotics

One physician mentioned that the protocol to admit any form of pneumonia from the ED attendance would increase the possibility of treating a COVID-19 related pneumonia as a bacterial pneumonia. The increase in antibiotic use could accelerate the development of antibiotic resistance.

#### Subtheme (2): less priority on the development of new antibiotics

Two senior physicians expressed concern that the priority placed on developing the COVID-19 vaccines had reduced work on the development new antibiotics.

*“All the pharmaceutical companies are trying to ramp up COVID[-19] production, and COVID[-19] vaccine production […], and the research and development into new antibiotics to tackle the problems of antibiotic resistance maybe pushed further down the line.” (Institution 4, Senior Consultant).*

### Decrease in antibiotic prescribing

Two subthemes, (1) Less consults for non-COVID-19 related URTI complaints, (2) An opportunity to educate patients that antibiotics are not necessary for a viral illness, emerged from the perception that the COVID-19 pandemic had led to a decrease in antibiotic prescribing.

#### Subtheme (1): less consults for non-COVID-19 related URTI complaints

Physicians felt that the change in local epidemiology has reduced the number of URTI consults and hence, less requirement for antibiotics. The focus of patients seeking care in the ED has also shifted to getting COVID-19 diagnostics rather than asking for antibiotics.

*“I think the goal of them coming to ED also have shifted, more on they wanted to get a swab test done. So, they don’t really ask for antibiotics.” (Institution 4, Senior Resident Physician)*

#### Subtheme (2): opportunity to educate patients that antibiotics are not necessary for a viral illness

Both junior and senior physicians felt that the COVID-19 pandemic had provided them with an opportunity to educate their patients on appropriate antibiotic use. They felt that patients had better knowledge that COVID-19 is a viral illness. One physician was also able to use the additional examination in the ED to explain to patients that their condition does not warrant antibiotics treatment.

*“I think COVID[-19] has taught a lot of people [physicians] that you know it is a viral illness and you don’t go around recommending antibiotics. Starting that piece of knowledge has actually gone out to a lot of common folks, common people in the street. When previously this is a piece of knowledge that they don’t know, [have] never bothered to know.” (Institution 4, Senior Consultant).*

### No change in antibiotic prescribing

Two subthemes, (1) COVID-19 does not change the standard management of URTI, (2) Baseline antibiotic prescribing was low prior to COVID-19, emerged from the perception that the COVID-19 pandemic has not changed the antibiotic prescribing practices. Some felt that they have not been prescribing antibiotics liberally pre-pandemic, hence, the pandemic would not change their antibiotic prescribing practices since COVID-19 is viral in nature.

*“I think in general, there’s no shift in the management for uncomplicated cases. Firstly, even for COVID[-19], there’s no [need] for antibiotics. So, we still give the patient symptomatic medications.” (Institution 3, Resident).*

### Integrated results

Five meta-inferences were made from the integration of the quantitative and qualitative analyses (Table 5). The meta-inferences are summarised below:

**Table 5:** Meta-inferences on the changes in Emergency Department Physicians’ attitudes and behaviour on antibiotic prescribing after data integration

	Meta-inferences	Quantitative data	Qualitative data
1	There is less pressure to prescribe antibiotics due to diminished patient demand for antibiotics and having more opportunities to educate patients on viral illnesses	High prescribers were more likely to succumb to pressure to prescribe antibiotics pre COVID-19 [OR: 1.74, 95% CI (1.12, 2.71)]. The pressure faced by high prescribers diminished during the COVID-19 pandemic [OR: 0.99, 95% CI (0.70, 1.41)] Regardless of COVID-19, high prescribers were more likely to think that patients who attend the ED are demanding [OR: 1.44, 95% CI (1.09, 1.89)] and that educating patients about antibiotic use will reduce their work pressure in handling patients with URTI [OR: 1.46, 95% CI (1.05, 2.02)]	Some ED physicians felt that the focus of URTI-related ED visits has shifted to getting a COVID-19 test rather than asking for antibiotics during the pandemic. The pandemic had also provided them with the opportunity to introduce the concept of viral illnesses to their patients
2	A higher proportion of ED physicians self-reported lower antibiotic prescribing rates during the COVID-19 pandemic but their perception of the overall outlook on antibiotic prescribing rates varied	ED physicians were twice as likely to self-report that they prescribed antibiotics for > 10% of URTI patients pre COVID-19 [OR: 2.12, 95% CI (1.32, 3.41)]. There was no difference in the perception of antibiotic over-prescribing between high and moderate prescribers regardless of COVID-19 [OR: 1.19, 95% CI (0.92, 1.54)] (the interaction term for this factor was dropped from the model)	Some physicians had the perception that more antibiotics were prescribed due to more diagnostic tests (X-rays) leading to over-diagnosis; some felt that there was no change in prescribing patterns due to COVID-19 due to its viral nature; some felt that the pandemic had given them the opportunity to reduce unnecessary antibiotic use
3	Physicians who were high antibiotic prescribers during the COVID-19 pandemic made less effort for prudent antibiotic prescribing as they were less concerned about antimicrobial resistance	There was no difference between high and moderate antibiotic prescribers pre COVID-19 in the effort to prescribe antibiotics prudently [OR: 1.10, 95% CI (0.73, 1.66)]. However, high prescribers were less likely to make an effort to prescribe antibiotics prudently during the COVID-19 pandemic [OR: 1.54, 95% CI (1.09, 2.18)]. Physicians concerned about antibiotic resistance would tend to make an effort to prescribe antibiotics prudently (the Likert item “I avoid prescribing antibiotics for patients with URTI because I am concerned about antibiotic resistance” was part of the behaviour-related factor "effort to prescribe antibiotics prudently") There was no significant difference in the distribution of this Likert item between high and moderate prescribers pre-COVID-19 (pre-COVID-19 mean (SD): 4.01 (0.78); during COVID-19 mean (SD): 4.22(0.65); *P* = 0.058), but high prescribers were significantly agreeing less to this statement during COVID-19 (pre-COVID-19 mean (SD): 3.68 (0.78); during COVID-19 mean (SD): 4.18 (0.68); *P* < 0.001).)	Many physicians expressed concern about (1) the acceleration of antibiotic resistance due to less priority on the development of new antibiotics and (2) the possible less prudent use of antibiotics due to COVID-19. Some of them mentioned that their concern about antimicrobial resistance had led them to prescribe antibiotics more prudently
4	COVID-19 did not change the factors that lowered the threshold for antibiotic prescribing	High prescribers were more likely to lower their threshold for antibiotic prescribing for patients who are immunocompromised, elderly, suspected of borderline bacterial infection, and have reattended the ED regardless of COVID-19 [OR: 1.83, 95% CI (1.37, 2.42)]. The COVID-19 interaction term was dropped as there were no significant differences in lowering the threshold for antibiotic prescribing pre- and post-COVID-19	Physicians would lower their antibiotic prescribing threshold for COVID-19 patients with poor premorbid or are immunocompromised
5	COVID-19 did not change the perception that the public's knowledge of antibiotics is poor	The factor "Insufficient patient education" was dropped from the logistic regression model as the variable did not contribute significantly to the model. There were no significant differences in perceptions among high/moderate prescribers and pre-or post-COVID-19	Physicians mentioned that despite improvement in the publics' awareness of antibiotic use in recent years, the public still lacked appropriate knowledge on antibiotic use. Therefore, the COVID-19 pandemic provided them with the opportunity to educate patients on appropriate antibiotic use if patients requested for antibiotics during the pandemic

#### Meta-inference 1

There is less pressure to prescribe antibiotics due to reduced patient demand for antibiotics and having more opportunities to educate patients on viral illnesses.

The quantitative data showed that high prescribers were more likely to perceive patients as demanding and educating them about antibiotic use will reduce their work pressure in handling URTI. Some physicians felt that the COVID-19 pandemic had eased the demand for antibiotics as the focus of URTI-related ED visits has changed from asking for antibiotics to getting a COVID-19 test. In addition, the pandemic had provided an opportunity for physicians to introduce the concept of viral illnesses to their patients and educate them on the appropriate management of such illnesses.

#### Meta-inference 2

Despite a higher proportion of ED physicians self-reporting lower antibiotic prescribing rates during the COVID-19 pandemic, their perception of antibiotic prescribing rates due to COVID-19 were varied.

The perception of overall antibiotic prescribing rates varied among physicians regardless of the COVID-19 pandemic (the interaction term was insignificant and dropped from the logistic regression model). Despite ED physicians self-reporting a lower rate of antibiotic prescribing during the COVID-19 pandemic, some physicians felt that antibiotic prescribing could have increased due to more chest radiographs. Some physicians felt that there was no change in prescribing patterns due to the viral nature of the disease, while others felt that antibiotic prescribing would decrease as the COVID-19 pandemic had enabled them to reduce unnecessary antibiotic prescribing.

#### Meta-inference 3

High antibiotic prescribers made less effort to prescribe prudently during the COVID-19 pandemic as they were less concerned about antimicrobial resistance.

There was no difference between high and moderate antibiotic prescribers in their efforts to prescribe antibiotics prudently pre-COVID-19 pandemic. However, high prescribers were less likely to make an effort to prescribe antibiotics prudently during the COVID-19 pandemic. Physicians concerned about antibiotic resistance would tend to make an effort to prescribe antibiotics prudently (the Likert item “I avoid prescribing antibiotics for patients with URTI because I am concerned about antibiotic resistance” was part of the behaviour-related factor "effort to prescribe antibiotics prudently"). There was no significant difference in the distribution of this Likert-scale item between high and moderate prescribers pre-COVID-19 (pre-COVID-19 pandemic mean (SD): 4.01 (0.78); during COVID-19 pandemic mean (SD): 4.22(0.65); *P* = 0.058), but high prescribers were significantly less likely to agree with this statement during the COVID-19 pandemic (pre-COVID-19 pandemic mean (SD): 3.68 (0.78); during COVID-19 pandemic mean (SD): 4.18 (0.68); *P* < 0.001).

The qualitative analyses showed that many physicians expressed concern about (1) the acceleration of antibiotic resistance due to less priority on the development of new antibiotics and (2) the possible less prudent use of antibiotics due to COVID-19. Some of them mentioned that their concern about antimicrobial resistance had led them to prescribe antibiotics more prudently. Therefore, physicians concerned about antimicrobial resistance would have prescribed fewer antibiotics (became moderate prescribers) during the COVID-19 pandemic.

#### Meta-inference 4

The COVID-19 pandemic did not change the factors that lowered the threshold for antibiotic prescribing.

High antibiotic prescribers were more likely to lower their antibiotic prescribing threshold for immunocompromised patients, the elderly, patients with a borderline bacterial infection, and patients who had reattended at the ED regardless of the COVID-19 pandemic (the interaction term was insignificant and dropped from the logistic regression model). During the COVID-19 pandemic, physicians would lower their antibiotic prescribing threshold for COVID-19 patients with poor premorbid or immunocompromised as they were concerned about secondary bacterial infections.

#### Meta-inference 5

The COVID-19 pandemic did not change physicians' perception that the public's knowledge of antibiotics is poor.

Although the factor of "Insufficient patient education" was dropped from the final logistic regression model, both high and moderate antibiotic prescribers had a high tendency to agree that the public knowledge of antibiotic use is poor (pre-COVID-19 mean (SD): 3.98 (0.75); during COVID-19 mean (SD): 3.85(0.80). A mean of above 3 indicates a general agreement with the Likert statement. Physicians also mentioned that despite improvement in the public's awareness of antibiotic use in recent years, the public still lacked appropriate knowledge on antibiotic use. Therefore, the COVID-19 pandemic provided them with the opportunity to educate patients on appropriate antibiotic use if patients requested for antibiotics during the pandemic.

## Discussion

Our study explored the changes in physicians’ attitudes and behaviours toward antibiotic prescribing for URTI in Singapore during the COVID-19 pandemic. The participants (of various designations) from four EDs (under three healthcare clusters) represented wide-ranging views on the topic. Data integration from in-depth interviews strengthened the interpretation of our findings from the questionnaire survey and provided plausible explanations for the phenomena observed.

Our findings showed a decrease in the proportion of ED physicians with self-reported high antibiotic prescribing rates during the COVID-19 pandemic compared with the pre-pandemic period. This observation concurs with other studies (in Hong Kong, Netherlands, Portugal) reporting a decrease in antibiotic prescribing trends in the outpatient settings [30–32]. Another US study reported that the outpatient and ambulatory setting prescribed fewer antibiotics during the COVID-19 pandemic due to milder severity and greater diagnostic certainty from the polymerase chain reaction (PCR) tests [33]. The decrease in antibiotic prescribing in Singapore likely stems from the decline in non-COVID-19 related URTI cases with the safe management measures and a shift in the purpose of ED visits during the pandemic. Although physicians prescribed fewer antibiotics during the COVID-19 pandemic, this trend may be unsustainable post-pandemic when the social distancing measures are relaxed. Hence, it is imperative to continue antimicrobial stewardship efforts post-pandemic.

A few physicians mentioned that the COVID-19 pandemic provided them with the opportunity to educate patients on the concept of viral illnesses. Such initiatives are essential to dispelling misinformation that overemphasises the role of antimicrobials in treating COVID-19 [34]. Given physicians’ poor impression of the public’s knowledge of antibiotic use and AMR, primary care physicians should also seize the opportunity to educate patients on the concept of viral illnesses should patients ask about using antimicrobials to treat COVID-19. Good public communication in Singapore has also helped educate patients on the proper management of COVID-19 and relieved ED physicians’ pressure to prescribe antibiotics [35].

Attitudes and behaviours ingrained in one’s practices are often difficult to change [36]. Physicians who remained as high antibiotic prescribers during the COVID-19 pandemic have fewer concerns about AMR. The lack of concern among this group of physicians widens the gap in the effort to prescribe antibiotics prudently compared with those concerned about AMR during the COVID-19 pandemic. Therefore, emphasis on medical student and continual physician education on AMR is necessary. Physicians with a lower threshold for the vulnerable population tend to have higher antibiotic use. The threshold on antibiotic use is a factor that is unaffected by the COVID-19 pandemic, as it is influenced by physicians’ confidence, experience, and diagnostic certainty in making the prescribing decision [37, 38]. As such, clinical decision support tools can potentially address physicians’ knowledge deficits and confidence in antibiotic prescribing, particularly the inexperienced physicians. The upside is that the pandemic did not further lower ED physicians’ antibiotic threshold for patients.

Despite the advantages of the mixed-methods approach in garnering deeper insights on our topic of interest, limitations exist in the study. First, the quantitative data collection period spanned multiple COVID-19 waves in Singapore. Hence, respondents’ attitudes and behaviours may have undergone multiple transient changes throughout the data collection. This limitation reflects the challenges in conducting research during the COVID-19 pandemic. Second, the physician survey was cross-sectional. Hence, we could not conduct longitudinal analyses to ascertain the specific factors influencing individual physicians in reducing antibiotic use pre-and during the COVID-19 pandemic. Third, we did not corroborate the self-reported antibiotic prescribing rates with the ED’s prescription data. Therefore, we could not ascertain the actual rates of antibiotic use (i.e., a physician attending to very few URTI cases, and many immunocompromised patients would have higher rates of antibiotic use). Nonetheless, as respondents were likely to have self-reported their prescribing rates the same way, any misclassification bias is likely towards the null. Lastly, there may be non-response bias as physicians who do not respond to our recruitment could have different antibiotic prescribing practices from our participants. We do not foresee this as a serious problem as we have achieved data saturation for our IDIs, and the protocols in the EDs would have prevented notable practice deviations.

Future work should assess the trends in antibiotic prescribing during the pandemic by examining the historical antibiotic prescribing data from electronic health records. Qualitative studies could also employ participatory research methods to develop and focus antimicrobial stewardship efforts on interventions acceptable to ED physicians.

## Conclusion

In conclusion, self-reported antibiotic prescribing rates for URTI at EDs decreased during the COVID-19 pandemic due to less pressure to prescribe antibiotics. The shift in focus reduced physicians’ pressure to prescribe antibiotics. However, the pandemic did not change factors that lower physicians’ threshold for antibiotic use. Since the pandemic provided some physicians with the opportunity to educate patients about viral respiratory illnesses, the lessons learnt from COVID-19 can be incorporated into the public and medical education in the war against AMR going forward. Antibiotic use should also be monitored post-pandemic to assess if the changes are sustained.

## Supplementary Information

**Additional file 1**. Additional tables.

## Data Availability

The final dataset is partially available on request.
