# Correction: A multi-institutional exploration of emergency medicine physicians’ attitudes and behaviours on antibiotic use during the COVID-19 pandemic: a mixed-methods study

**DOI:** 10.1186/s13756-023-01255-7

**Published:** 2023-06-01

**Authors:** Zhilian Huang, Evonne Tay, Win Sen Kuan, Ling Tiah, Yanyi Weng, Hann Yee Tan, Eillyne Seow, Li Lee Peng, Angela Chow

**Affiliations:** 1 Department of Preventive and Population Medicine, Office of Clinical Epidemiology, Analytics, and Knowledge [OCEAN], Tan Tock Seng Hospital, 11 Jalan Tan Tock Seng, Singapore, 308433 Singapore; 2 Department Emergency Medicine, National University Hospital, Singapore, Singapore; 3 Department of Surgery, Yong Loo Lin School of Medicine, National University of Singapore, Singapore, Singapore; 4 Accident & Emergency Department, Changi General Hospital, Singapore, Singapore; 5 Department Emergency Medicine, Tan Tock Seng Hospital, Singapore, Singapore; 6 Acute and Emergency Care Department, Khoo Teck Puat Hospital, Singapore, Singapore; 7 Lee Kong Chian School of Medicine, Nanyang Technological University, Singapore, Singapore; 8 Infectious Diseases Research and Training Office, National Centre for Infectious Diseases, Singapore, Singapore; 9 Saw Swee Hock School of Public Health, National University Singapore, Singapore, Singapore

Antimicrobial Resistance & Infection Control (2023) 12:24

10.1186/s13756-023-01230-2

The original article [1] contained an error in attribution and display of affiliation #8 which has since been corrected.
