# Supplementary material for: A multi-institutional exploration of emergency medicine physicians’ attitudes and behaviours on antibiotic use during the COVID-19 pandemic: a mixed-methods study
Source: Antimicrob Resist Infect Control. 2023 Mar 29;12:24. doi: 10.1186/s13756-023-01230-2 (PMC10057674; doi:10.1186/s13756-023-01230-2)
Supplement: Supplementary file 1 — Additional file 1. Additional tables. [file 13756_2023_1230_MOESM1_ESM.docx]

**Supplementary Materials**

**Table S1a. Attitudes Likert-scale items in the survey**

|  | **Variables in factor analysis** | **Variance explained** |  |
| --- | --- | --- | --- |
|  | All factors included | **46.24** | **-** |
| **Item No.** | **Variables excluded in factor analysis** | **Variance explained after dropping factors** | **Comments** |
| C9 | This hospital will support my clinical decisions in managing patients with URTI. | 47.45 | Dropped |
| C13 | Educating the public on appropriate antibiotic use will reduce my work pressure in handling URTI patients. | 48.17 | Dropped |
| C20 | In this ED, the management puts patient safety at the same level of importance as meeting work productivity. | 49.56 | Dropped |
| C16 | Patient education on antibiotic use is the responsibility of the ED. | 51.17 | Dropped |
| C17 | Patient education on antibiotic use is the responsibility of primary care clinics. | 53.56 | Dropped |
| C1 | I am confident of my antibiotic prescribing decisions for patients with URTI. | 55.73 | Dropped |
| C2 | Patients should take antibiotics for URTI because the risks (e.g. drug allergy, side effects etc.) are low. | 58.45 | Dropped |
| C3 | Patients attending this ED are demanding about their treatment (including requesting for antibiotics for URTI). | 60.59 | Dropped |
| C8 | This hospital will support my clinical decisions in managing patients with URTI. | 63.59 | Dropped |
| C11 | I think an antibiotic prescribing surveillance system that monitors the rate of antibiotic prescribing within the ED will help me reduce unnecessary prescribing of antibiotics. | 66.47 | Dropped |
| **Item No.** | **Variables included in factor analysis** | **Cronbach's alpha** | **Proposed factor name** |
| C4 | I think antibiotics are over-prescribed in this ED. | 0.685 | Perception of antibiotic over-prescribing in the ED |
| C6 | My antibiotic prescribing decisions for URTI patients are different from my seniors (i.e. think about seniors who have guided you irrespective of your current appointment). |  |  |
| C7 | My antibiotic prescribing decisions for URTI patients are different from my peers. |  |  |
| C5 | I think I should cut down on the amount of antibiotics that I prescribe. |  |  |
| C22 | When I take the time to communicate about patient safety problems in this ED, there is appropriate follow-up. | 0.664 | Organization safety culture |
| C23 | In this ED, staff speak up freely if they see something that may negatively affect patient care. |  |  |
| C21 | This ED takes time to identify and assess risks to ensure patient safety. |  |  |
| C10 | I think a clinical decision support tool will be useful to guide my decision on antibiotic prescribing for URTI patients. | 0.666 | Clinical decision support tool for antibiotic prescribing |
| C19 | I would use an app (if available) to help me make antibiotic prescribing decisions for URTI patients. |  |  |
| C14 | Posters are useful in educating patients about antibiotic use and resistance. | 0.763 | Patient education on antibiotics use and antibiotics resistance |
| C15 | Digital platforms (e.g., mobile app, videos) are useful in educating patients about antibiotic use and resistance. |  |  |
| C12 | Patient education on antibiotic use is currently sufficient in Singapore. | 0.696 | Insufficient patient education |
| C18 | Public knowledge on antibiotic use is poor. |  |  |

Item numbers are listed in accordance with the order they are dropped from the PCA and grouped according to their factors.

**Table S1b: Behaviour Likert-scale items in the survey**

|  | **Variables in factor analysis** | **Variance explained** |  |
| --- | --- | --- | --- |
|  | All factors included | **53.20** | **-** |
| **Item No.** | **Variables excluded in factor analysis** | **Variance explained after dropping factors** | **Comments** |
| D14 | I will prescribe antibiotics for URTI patients who have poor social support (i.e. lack of capacity to return for a follow-up). | 53.46 | Dropped |
| D15 | I will prescribe antibiotics if an URTI patient declines further investigations even if he/she does not need them. | 53.94 | Dropped |
| D27 | I will discuss with the senior doctors in the department, if I am uncertain about the management of an URTI patient. | 54.73 | Dropped |
| D9 | I will prescribe antibiotics to ensure a bacterial infection is not missed. | 55.37 | Dropped |
| D29 | I will use a point-of-care test (if available) to help me differentiate between viral and bacterial URTIs. | 56.97 | Dropped |
| D7 | I will prescribe antibiotics if I am concerned about the risk of bacterial complications from a viral URTI. | 58.30 | Dropped |
| D22 | I will prescribe antibiotics for URTI patients, if they have waited a long time for medical consultation. | 58.79 | Dropped |
| D28 | I rely on international guidelines (e.g., Australian Therapeutic Guidelines, Centers for Disease Control and Prevention Guidelines) for antibiotic prescribing decisions in general. | 61.52 | Dropped |
|  | Horn's parallel analysis indicated 4 factors | 56.48 | Dropped |
| D6 | I have difficulty determining whether antibiotics are needed for patients with URTI. | 58.69 | Dropped |
| D8 | I depend on diagnostic tests (e.g. FBC, chest X-ray) to decide on whether to prescribe antibiotics for patients with URTI. | 61.09 | Dropped |
| D16 | I will prescribe antibiotics for URTI patients to keep on standby, in case they need them later (e.g., travelling to another country, condition deteriorates). | 63.05 | Dropped |
| D24 | My antibiotic prescribing decision is influenced by what I was taught at this hospital. | 65.10 | Dropped |
| **Item No.** | **Variables included in factor analysis** | **Cronbach's alpha** | **Proposed factor name** |
| D18 | I will prescribe antibiotics for URTI patients if they or their relatives demand for them. | 0.922 | Pressure to prescribe antibiotics (Patient attributed) |
| D20 | To maintain good relationships with patients, I will prescribe antibiotics for patients with URTI who demand for them. |  |  |
| D10 | I will prescribe antibiotics for URTI patients who demand for them when I have many patients waiting to be attended to. |  |  |
| D17 | I will prescribe antibiotics for URTI patients if they or their relatives ask for them. |  |  |
| D21 | I will prescribe antibiotics for URTI patients who are anxious about their condition |  |  |
| D23 | I will prescribe antibiotics for URTI patients if I think patients or their relatives expect them. |  |  |
| D1 | At this ED, I usually treat and manage patients with uncomplicated URTI symptomatically | 0.707 | Effort to prescribe antibiotics prudently due to concerns about antibiotic resistance |
| D3 | I avoid prescribing antibiotics for patients with URTI because I am concerned about antibiotic resistance. |  |  |
| D4 | I will explain to patients with URTI why they do not need antibiotics if they ask for them. |  |  |
| D2 | I rely only on my clinical judgement in treating and managing patients with URTI. |  |  |
| D5 | I am confident of convincing patients who do not need antibiotics to not take them. |  |  |
| D12 | I will prescribe antibiotics for URTI patients with comorbidities who are immunocompromised. | 0.740 | Lowered threshold for antibiotics prescribing |
| D11 | I will prescribe antibiotics for elderly patients (aged >65 years) with URTI. |  |  |
| D13 | I will prescribe antibiotics for URTI patients who re-attend at this ED for the same respiratory condition. |  |  |
| D10 | I will prescribe antibiotics in the situation of borderline diagnosis of bacterial URTI. |  |  |
| D26 | I will re-examine my previous antibiotic prescribing decision for URTI patients if it was different from my seniors. | 0.874 | Peer influence on antibiotic prescribing |
| D25 | I will re-examine my previous antibiotic prescribing decision for URTI patients if it was different from my peers. |  |  |

Item numbers are listed in accordance with the order they are dropped from the PCA and grouped according to their factors.

**Table S2: AIC and BIC Model for variables included**

| **Model** | **Variables included in the model** | **AIC** | **BIC** |
| --- | --- | --- | --- |
| M1 | Attitudes-F1 + Attitudes-F2 + Attitudes-F3 + Attitudes-F4 + Attitudes-F5 + Behaviour-F1 + Behaviour-F2 + Behaviour-F3 + Behaviour-F4 + Institution + Physician designation (binary) + Place of medical education (binary) + Years of practice (binary) + COVID + Attitudes-F1*COVID + Attitudes-F2*COVID + Attitudes-F3*COVID + Attitudes-F4*COVID + Attitudes-F5*COVID + Behaviour-F1*COVID + Behaviour-F2*COVID + Behaviour-F3*COVID + Behaviour-F4*COVID | 576.63 | 687.87 |
| M2 | M1 - Place of medical education (binary) - Physician designation (binary) | 574.31 | 677.13 |
| M3 | M2 - Behaviour-F4*COVID - Behaviour-F3*COVID - Years of practice (binary) - Attitudes-F1*COVID - Attitudes-F4*COVID - Attitudes-F2*COVID - Attitudes-F3*COVID - Attitudes-F2 - Attitudes-F4 - Attitudes-F5*COVID - Attitudes-F5 - Institution | 559.54 | 602.44 |
| M4 | M3 + Attitudes-Likert C1 + Attitudes-Likert C2 + Attitudes-Likert C3 + Attitudes-Likert C13 | 535.15 | 595.18 |
| M5 | M4 + Behaviour-Likert D16 + Behaviour-Likert D22 + Behaviour-Likert D27 | 531.01 | 603.77 |
| **M6 (Final model)** | **M5 - Attitudes-F3 - Attitudes-F4** | **529.79** | **598.27** |

AIC, Akaike information criterion; BIC, Bayesian information criterion

**Physician designation (binary):** Junior (Medical Officer/ Resident/ (Senior) Resident Physician) or Senior (Senior Resident/ Staff Registrar/ Principal Resident Physician/ Associate Consultant/ Consultant/ Senior Consultant)

**Place of medical education (binary):** Local (Singapore) or Overseas (Others)

**Years of practice (binary):** ≤5 Years or >5 Years

**Table S3: Qualitative interview questions (semi-structured guide)**

| **Qualitative Interview Guide** |
| --- |
| **Behaviour and attitude towards the treatment of URTI (Pre-COVID-19)** |
| URTI is a broad and non-specific diagnosis in the ED. Putting COVID-19 aside, can you share with me your definition of URTI in general? |
| Next, I would like to learn about your opinions regarding the treatment of URTI. Putting COVID-19 aside, what do you think is the common practice of treating URTI at this ED? |
| Under what circumstances would you prescribe antibiotics for the treatment of URTI? |
| Are there non-clinical factors that may play a part in your decision to prescribe antibiotics? |
| **Behaviour and attitude towards the treatment of URTI (During COVID-19)** |
| Over the COVID-19 period in this ED, have you noticed any changes in institutional or departmental practices on treating URTI? Please elaborate. |
| What would your treatment plan be for suspected COVID-19 patients?  *Probe: Would you include antibiotics in the treatment plan? Why?* |
| **Behaviour and attitude towards antibiotic resistance** |
| Were your antibiotic prescribing practices affected by your views on antibiotic resistance? |
| How do you think the COVID-19 pandemic will affect the development of antibiotic resistance? What makes you say so? |
| Were your antibiotic prescribing practices for COVID-19 suspects/cases affected by your concern on antibiotic resistance? |
